# Supplementary material for: CarbaDetector: a machine learning model for detecting carbapenemase-producing Enterobacterales from disk diffusion tests
Source: Nat Commun. 2025 Nov 14;16:10023. doi: 10.1038/s41467-025-66183-z (PMC12618456; doi:10.1038/s41467-025-66183-z)
Supplement: Supplementary file 1 — Supplementary Information [file 41467_2025_66183_MOESM1_ESM.pdf]

CarbaDetector: A Machine Learning Model for Detecting Carbapenemase-Producing Enterobacterales from Disk Diffusion Tests

Linea Katharina Muhsal, Cansu Cimen, Janko Sattler, Lisa Theis, Oliver Nolte, Laurent Dortet, Rémy A. Bonnin, Adrian Egli, Axel Hamprecht

Supplementary information

Methods

Statistical analysis, classification, and development of an app for carbapenemase detection

Analysis was performed using R Statistical Software[1]. The packages tidyverse (2.0.0), tidyr (1.3.1), caret (7.0.1), tree (1.0.44), rpart (4.1.24), missRanger (2.6.1) and randomForest (4.7.1.2) were used for model creation[2-8], ggplot2 (4.0.0) was used for plotting[9]. Data was centered and scaled prior to analysis. Isolates that were present in the internal dataset less than 10 times were denoted as “Other”. Isolates in the external datasets that had no corresponding species in the internal dataset were denoted as “Other”.

Furthermore, we developed CarbaDetector, an easy to use web-app employing up to eight inhibition zone diameters provided by the user to predict the presence or absence of carbapenemase production based on a random forest model. The web-app was built using shiny[10] (1.11.1).

Results

Strain collections

Internal dataset (n=385)

Six species constituted 88.8 % of the isolates, namely *Klebsiella pneumoniae* (29.6 %), *E. coli* (17.9 %), *Citrobacter freundii* (16.9 %), *Enterobacter cloacae* (16.6%), *P. mirabilis* (4.2%), and *Serratia marcescens* (3.6 %). Of all 385 isolates, 238 (61.8 %) were carbapenemase producers, 147 (38.2 %) were carbapenemase-negative. The most frequent carbapenemase group present was *bla*$_{\text{OXA-48-like}}$ (n =

111, 46.6 %), followed by *bla*$_{VIM}$ (n=72, 30.3 %), *bla*$_{NDM}$ (n = 26, 10.9 %), *bla*$_{IMP}$ (n = 12, 5.0 %), *bla*$_{KPC}$ (n = 8, 3.4 %), *bla*$_{IMI}$ (n = 7, 2.9 %), and others (n = 7, 2.9 %). The most represented Ambler-class was class D (n = 111, 46.6 %), followed by class B (n = 110, 46.2 %), and class A (n = 17, 7.1 %).The dataset is available as supplementary data 1.

External dataset A (n=282)

Five species constituted 95.4 % of the isolates, namely *K. pneumoniae* (47.2 %), *E. coli* (26.2 %, *Enterobacter cloacae complex* (16.3 %), *C. freundii* (2.8 %), and *M. morganii* (2.8 %). Of 282 isolates, 80 (28.4 %) were carbapenemase producers. The most represented Ambler class was Class D (n = 40, 50.0 %), with 6/40 isolates harboring an additional Class B and 1/40 an additional Class A carbapenemase, followed by Class B (n = 22, 27.5 %) and Class A (n = 18, 22.5 %). Isolates were characterized by whole genome sequencing and/or PCR for carbapenemase genes, followed by Sanger sequencing. The dataset is available as supplementary data 2.

External dataset B (n=518)

External dataset B was previously published by Duque et al.[11]. It is available as supplementary data 3.

Models

Table S1: Importance parameters for the final random forest model CarbaDetector. Combinations of two abbreviations (such as temcza) denote the difference between the inhibition zone diameters of these two antibiotics.

variable	importance
imipenem_relebactam	100
temocillin	70,88935738
imipenem	67,2013686
czatem	58,2989057
czaimr	49,53810948
ceftolozan_tazobactam	46,04408211
etpimr	42,96966737
czaimi	41,74839707
meropenem	40,5707028
etpimi	39,3142731
meropenem_vaborbactam	39,08394011
etpmem	38,09370631
czamev	38,07423094
ceftazidim_avibactam	35,21216278
czamem	33,71560663
etpmev	30,67901859
imrmem	27,42460961
etptem	27,27103347
czaetp	24,76290274
memmev	24,62771791
imiimr	24,26221805
cetaimr	20,86475693
imitem	19,24676749
cetatem	18,77235493
memtem	16,67364457
imrmev	16,50505961
ertapenem	14,02457415
imrtem	13,96186704
species	13,84462897
mevtem	13,64171018
cetaetp	12,98461663
cetaimi	9,936930369
imimem	8,946132898
cetamem	4,621530366
czaceta	2,849833958
imimev	2,242444694
cetamev	0

Hyperparameters of the CarbaDetector model

mtry = 4, chosen from 4, 6, 8, and 10.

Comparison of species-specific performance

Species-specific performance was determined for *K. pneumoniae* and *E. coli* isolates. When assessing CarbaDetector results for *K. pneumoniae* isolates, a sensitivity of 96.8 % and a specificity of 92.3 % was achieved. Training of a species-specific model results in 98.4 % sensitivity and 88.5 % specificity. CarbaDetector results for *E. coli* yielded a sensitivity of 93.3 % and a specificity of 87.5 %. The training of a *E. coli*-specific model resulted in 97.8 % sensitivity and 70.8 % specificity.

False negative isolates predicted on original dataset by CarbaDetector

Table S2: Disk diffusion diameters (mm) and broth microdilution MICs (mg/L) for the eight false negative isolates predicted by CarbaDetector.

Isolate		B7-26	B6-77	Z-01393	B2-3	B4-41	B7-7	B5-4	B3-7
Species		*K. pneumoniae*	*E. hormaechei*	*S. marcescens*	*E. coli*	*K. pneumoniae*	*E. coli*	*E. cloacae*	*E. coli*
Meropenem	DDT	20	17	28	27	16	27	30	26
	BMD	2	4	0.5	0.5	8	0.12	0.06	0.5
Imipenem	DDT	25	17	27	28	18	24	26	24
	BMD	0.5	2	2	0.25	8	0.5	0.25	1
Ertapenem	DDT	9	8	23	19	13	23	24	16
	BMD	8	16	0.5	2	8	0.25	0.125	2
Meropenem-vaborbactam	DDT	21	19	32	30	24	29	31	29
	BMD	2	1	0.5	0.5	0.03	0.06	0.03	0.25
Imipenem-relebactam	DDT	26	24	27	29	29	26	28	26
	BMD	0.5	1	2	0.25	0.25	0.5	0.125	1
Ceftazidime-avibactam	DDT	20	21	17	25	18	24	25	20
	BMD	2	1	8	0.25	2	0.12	0.25	1
Ceftolozan-tazobactam	DDT	9	22	18	28	11	23	26	14
	BMD	>128	1	32	0.5	128	1	0.5	64
temocillin	DDT	10	20	15	21	13	14	27	6
	BMD	64	8	32	16	32	32	4	64
Carbapenemase		OXA-244	VIM-1	IMP-13	VIM-1	KPC-3	OXA-244	VIM-1	OXA-181

DDT =Disk diffusion, BMD= Broth Microdilution

References

1 R: A Language and Environment for Statistical Computing (R Foundation for Statistical Computing, Vienna, Austria, 2024).

2 Wickham, H. *et al.* Welcome to the Tidyverse. *Journal of open source software* **4**, 1686 (2019).

3 Wickham, H. & Wickham, M. H. Package 'tidyr'. *Easily Tidy Data with'spread'and'gather ()'Functions* (2017).

4 Kuhn, M. *et al.* Package 'caret'. *The R Journal* **223**, 48 (2020).

5 Ripley, B. & Ripley, M. B. Package 'tree'. (2023).

6 Therneau, T., Atkinson, B., Ripley, B. & Ripley, M. B. Package 'rpart'. *Available online: cran. ma. ic. ac. uk/web/packages/rpart/rpart. pdf (accessed on 20 April 2016)* (2015).

7 Breiman, L. Random Forests. *Machine Learning* **45**, 5-32 (2001). https://doi.org/10.1023/A:1010933404324

8 Mayer, M. missRanger: Fast Imputation of Missing Values. (2025). <https://github.com/mayer79/missRanger>.

9 Wickham, H. ggplot2. *Wiley interdisciplinary reviews: computational statistics* **3**, 180-185 (2011).

10 shiny: Web Application Framework for R v. R package verion 1.9.1 (2024).

11 Duque, M., Bonnin, R. A. & Dortet, L. Evaluation of the French novel disc diffusion-based algorithm for the phenotypic screening of carbapenemase-producing Enterobacterales. *Clinical Microbiology and Infection* **30**, 397.e391-397.e394 (2024). https://doi.org/https://doi.org/10.1016/j.cmi.2023.12.003
