# Supplementary material for: CarbaDetector: a machine learning model for detecting carbapenemase-producing Enterobacterales from disk diffusion tests
Source: Nat Commun. 2025 Nov 14;16:10023. doi: 10.1038/s41467-025-66183-z (PMC12618456; doi:10.1038/s41467-025-66183-z)
Supplement: Supplementary file 3 — Description of Additional Supplementary Files [file 41467_2025_66183_MOESM3_ESM.pdf]

Description of Additional Supplementary Files

Title: Supplementary Data 1

Description: Internal dataset used for the development of the model, including inhibition zone measurements, species and presence of carbapenemases for 385 isolates and the prediction outcomes of EUCAST, CA-SFM algorithm as well as several algorithms detailed in Table 2

Title: Supplementary Data 2

Description: External dataset A used for the validation of CarbaDetector, including inhibition zone measurements, species and presence of carbapenemases for 282 isolates and the prediction outcomes of EUCAST, CA-SFM algorithm, as well as CarbaDetector.

Title: Supplementary Data 3

Description: External dataset B used for the validation of CarbaDetector, including inhibition zone measurements, species and presence of carbapenemases for 518 isolates and the prediction outcomes of EUCAST, CA-SFM algorithm, as well as CarbaDetector
